# SF-6D utility values for the better- and worse-seeing eye for health states based on the Snellen equivalent in patients with age-related macular degeneration

**DOI:** 10.1371/journal.pone.0169816

**Published:** 2017-02-22

**Authors:** Martijn S. Visser, Sankha Amarakoon, Tom Missotten, Reinier Timman, Jan J. Busschbach

**Affiliations:** 1 Rotterdam Ophthalmic Institute, Rotterdam, The Netherlands; 2 Erasmus Medical Center, Department of Psychiatry, section Medical Psychology and Psychotherapy, Rotterdam, the Netherlands; Tohoku University, JAPAN

## Abstract

**Objective:**

Economic evaluations in wet age-related macular degeneration (ARMD) is hampered as often utility values for solely one eye are used, mostly the better-seeing eye (BSE). Moreover, frequently chosen methods rely on patient values and/or disease specific measures, while economic evaluations prefer generic quality of life (QoL) measures based on societal preferences. The generic QoL utility instrument EQ-5D has shown to be insensitive for differences in visual acuity. The aim of this study was therefore to provide societal utility values, using the generic SF-6D, for health states acknowledging both BSE and worse-seeing eye (WSE).

**Methods:**

SF-6D utility values of 191 ARMD patients (≥65 years) with 153 follow-up measures at 1 year were used to fill health states defined by the combination of BSE and WSE using Snellen equivalents; no visual loss (≥20/40), mild-moderate (<20/40–>20/200) and severe (≤20/200).

**Results:**

QoL utilities were estimated for the SF-6D, ranging from 0.740 for ARMD patients without visual loss to 0.684 for patients with a combination of mild-moderate visual loss in their BSE and severe visual loss in their WSE.

**Conclusion:**

Societal utility values are provided for ARMD patients using the generic QoL instrument SF-6D for visual acuity health states based on both BSE and WSE. The range of the values is smaller than previous elicited utilities with the disease-specific VisQoL. Besides, the utility values are placed on a more realistic position on the utility scale, and SF-6D utility values avoid the problem associated with the interpretation of disease-specific utility values.

## Introduction

Age-related macular degeneration (ARMD) is the leading cause of severe vision loss and legal blindness among people over the age of 50 years in Western countries [1, 2]. Health economic evaluations in ARMD often assume that an outcome of interventions in the worse seeing eye (WSE) can be valued, in terms of quality of life (QOL), as if it was an outcome in the better seeing eye (BSE). Thus one assumes that an increase in visual acuity in the WSE has the same utility gain as an increase in visual acuity of the BSE. This assumption seems unlikely, as the WSE is assumed to be dominated by the BSE. On the other hand, economic evaluations sometimes do not even value the WSE at all, and assume that an increase in visual acuity in the WSE results in no utility gain, unless in improves beyond the BSE. This assumption seems also unlikely as this neglects the effect of loss of depth perception and loss of visual field. Therefore changes in the WSE, improvements or deteriorations, is expected to have a smaller influence in visual acuity than changes in the BSE, but should not be set to zero. Using utility of the BSE for effective interventions in the WSE might therefore lead to an overestimation of the benefit of interventions in ARMD, where not using them might lead to an underestimation of the benefit. The problem originates from using utility values based on a cross sectional sample, in which the health states were defined on the basis of the BSE [3, 4]. However, in clinical practice 60 to 72% of the eyes treated are WSEs [5, 6].

Several attempts have been made to overcome this flaw. One way is to use utilities valued by patients instead of utilities from the general public. However, in health economics values from the general public are preferred over patient values [7]. Finger et al. were the first to provide societal utilities for health states of visual acuity defined as the visual acuity in both the WSE and the BSE [8]. Their first attempt to do so failed, as a standard utility instrument, namely the EuroQol-5 dimensions (EQ-5D), seemed not sensitive enough. They were more successful using a disease-specific utility instrument, the Vision and Quality of Life Index (VisQoL) measuring disease-specific QoL. Because QoL instruments measure the subjective burden experienced by an individual, the utilities found were related to WSE and BSE eyes combined. After classifying the patients by their WSE and BSE visual acuity, Finger et al. could relate the utilities to visual acuity in both eyes. Finger et al. claimed that they had obtained utilities that could be used in health economic evaluation, notably Quality Adjusted Life Years (QALYs). However, it is still a discussion if the values of disease-specific instrument can be considered valid values for the estimation of QALYs. That is because values of the different stages of the ‘disease’ are measured in isolation of co-morbidity, as shown in Table 2 of the publication of Finger et al., which presents utilities for various stages of visual acuity between .95 and .85, which is above the average utility of the general population that is usually between .90 and .80 [9]. It is not clear how these utilities in isolation are related to utilities that were elected in the presence of co-morbidity issues and it is unknown whether they are on the same scale [10–12]. Moreover, there are indications that disease-specific instrument tend to overestimate effects of the chosen morbidity, because respondents who provide the utilities focused too much on the chosen morbidity [13].

A solution to all this may be the use of generic utility instruments. Yet, the most used generic QoL instrument, the EQ-5D, was not significant related to difference in visual acuity in both eyes [14, 15]. Alternative generic instruments that can be used are the HUI, the AQoL-7D and the short form 6-D (SF-6D), which is based on the popular SF- 36 [16–18]. The SF-6D showed to be able to differentiate between patients with ARMD [14, 15, 19], and is found to be more sensitive in mild health states than the EQ-5D [20].

In health economics, models are built on health states defined in the terms of the primary clinical outcome. In ARMD that would be visual acuity measured using ‘Snellen charts’ in a clinical settings or Early Treatment of Diabetic Retinopathy Study (ETDRS) charts in a research settings.[21] The aim of this study is to link SF-6D utilities to health states of visual acuity of the BSE and the WSE combined, defined in terms of the Snellen charts and the ETDRS charts.

## Methods

### Study design

The data in this analysis was used from a randomized controlled trial comparing three treatment regimens of bevacizumab (Avastin) for the treatment of ARMD [22]. In this trial a total of 191 ARMD patients, 65 years of age or older who had a visual acuity of 20⁄200 to 20⁄20 Snellen equivalents in the treatment eye, were randomized to treatment regimens of every 4, 6 or 8 weeks. The bevacizumab treatment consisted of a dose of 1.25 mg in a 0.05-ml solution. Treatment was continuous for one year, with visual acuity and QoL measures during their hospital visits at baseline and one year later. Written informed consent was obtained from all participants. The study was approved by the Erasmus Medical Research Ethics Committee (MEC-2007-254) and was registered in the Dutch Trial Register (NTR 1174). No difference in visual acuity [22] nor in QoL [23] was found between the three treatment regimens.

### Main outcome measures

Visual acuity in the treatment eye was measured with the Early Treatment Diabetic Retinopathy Study (ETDRS) chart by the letter-by-letter scoring method [24]. The Snellen charts were used in the non-treatment eye. The outcome of both charts were divided in no visual loss (≥20/40), mild-moderate visual loss (<20/40–>20/200) and severe visual loss (≤20/200). The two cut-offs chosen in line with a previous study [25]. In that study the cut-off of 20/40 was related to ‘legal driving vision’ and the 20/200 cut-off to ‘legal blindness’. The latter cut-off correspond with the exclusion criterion for the treatment eye. The two cut-offs together with the differentiation between BSE and WSE define 6 health states, of which one is excluded (BSE worse than 20/200).

Two instruments to measure QoL were used; one generic utility instrument, the SF-6D, and one disease-specific questionnaire, the NEI VFQ-39. A Dutch value set to compute utilities for the SF-6D is currently not available, and therefore the UK value sets was used [16, 17]. NEI VFQ-39 consists of a 25-item base set of questions and a supplement of 14 additional items measuring vision-related QoL, which can be summarized into a ‘total component score’; range 0–100 [26]. The NEI VFQ-39 will provide information whether and how the health states differ, when measured with a sensitive disease-specific instrument. Several studies proposed a 10-point difference to be minimal clinically important [27–29]. Note that this NEI VFQ-39 total component score is not a ‘utility score’ that can be used in QALY-analysis.

### Data analysis

#### Better- and worse-seeing eye

Better- and worse-seeing eyes were determined by the difference in letters. When baseline visual acuity in both eyes was equal or higher than 50 letters (20/100), a 5 letter difference was used as a minimal difference threshold to distinguish a BSE from a WSE [21, 30]. With a lower visual acuity, a minimal difference of 10 letters was established. When the minimal difference between the better- and worse-seeing eye did not met the criteria above, equal visual acuity was assumed.

#### Statistical analyses

Descriptive statistics analyses were performed. The observed utility means were displayed using the three health states for BSE and WSE; no visual loss (≥20/40), mild-moderate visual loss (<20/40–>20/200) and severe visual loss (≤20/200). To account for the dependency of the two measures per patient and to straighten out possible inconsistencies due to illogical orderings, the utilities of the SF-6D and total component score of the NEI VFQ-39 were also analyzed with multilevel regression analyses. Patients formed the upper level of that analysis, the repeated measures the lower level. Included covariates were: dummies reflecting mild-moderate vision in the BSE, mild-moderate vision in the WSE, severe vision problems in the WSE, gender, and age. The difference between the baseline and follow-up was not applied as a covariate as that is not of interest for the estimation of the utilities.

## Results

### Descriptive statistics

Characteristics of the included patients are shown in Table 1. Of the 191 patients enrolled at baseline, 3 patients were excluded from analyses because visual acuity was evaluated in only one eye. In total 39% (n = 73) of the patients were treated in the BSE against 48% (n = 91) patients in the WSE. For the remaining 13% (n = 24) equal visual acuity was assumed. Patients treated in the BSE had lower values compared to patients treated in the WSE on both SF-6D (.68 vs .74) and NEI VFQ (56 vs 75) at baseline. Patients treated in the BSE were slightly older than those treated in the WSE, 81 and 77 years respectively.

**Table 1:** Baseline characteristics relevant to patient-reported outcomes and visual acuity distribution according to better- or worse-seeing eye at baseline.

	Study eye		
	BSE	WSE	BSE = WSE
	(n = 73)	(n = 91)	(n = 24)
Mean age, years (SD)***	81.1 (5.4)	77.4 (6.7)	75.9 (6.7)
Male gender, n (%)	24 (33)	31 (34)	7(29)
VA BSE, Snellen equivalent, N (%)			
>20/40	33 (45)	80 (88)	16^1^(67)
20/40-20/80	29 (40)	11 (12)	7 (29)
20/80-20/200	11 (15)	0	1 (4)
VA WSE, Snellen equivalent, N (%)			
>20/40	9 (12)	28 (31)	15 (63)
20/40-20/80	4 (5)	38 (42)	8^1^(33)
20/80-20/200	11 (15)	25 (27)	1 (4)
20/200-20/400	14 (19)	0	0
<20/400	35 (48)	0	0
BSE VA at 2 meter, mean (SD)***	.34 (.23)	.12 (.16)	.23 (.25)
WSE VA at 2 meter, mean (SD)***	1.48 (.89)	.48 (.25)	.27 (.25)
SF 6D, mean (SD)**	.683 (.095)	.740 (.128)	.746 (.095)
NEI VFQ-25, mean (SD)***	56.37 (17.22)	75.14 (17.52)	75.22 (15.02)

** p < 0.01.

*** p <0.001.

^1^ In the same eye group, one person fell in different categories.

Abbreviations: BSE = better seeing eye; WSE = worse seeing eye; VA = visual acuity; SD = standard deviation.

**Table 2:** Observed means of SF-6D and NEI-VFQ and numbers per health state.

		WSE			
	BSE	No visual loss	Mild-moderate	Severe	Overall
		Mean (SD; N)	Mean (SD; N)	Mean (SD; N)	Mean (SD; N)
**SF-6D**	No visual loss	.755 (0.114; 92)	.727 (0.138; 88)	.717 (0.125; 44)	.736 (0.127; 224)
	Mild-moderate		.745 (0.110; 38)	.668 (0.114; 47)	.702 (0.118; 85)
	Overall	.755 (0.114; 92)	.732 (0.130;126)	.691 (0.121;91)	.727 (0.125; 309)
**NEI-VFQ**	No visual loss	82.4 (11.9; 98)	74.8 (17.1; 100)	67.1 (17.6; 50)	76.2 (16.3; 248)
	Mild-moderate		58.9 (16.1; 41)	49.3 (14.1; 52)	53.5 (15.7; 93)
	Overall	82.4 (11.9; 98)	70.2 (18.3; 141)	58.0 (18.2; 102)	70.1 (19.1; 341)

Abbreviations: BSE = better seeing eye; WSE = worse seeing eye; sd = standard deviation

no visual loss (≥20/40), mild-moderate visual loss (<20/40–>20/200) and severe visual loss (≤20/200).

After one year of treatment one patient was excluded from analyses as visual acuity was evaluated in only one eye and a total of 34 patients dropped out of the study: eighteen patients dropped out as a result of non-compliance, seven because of serious adverse events, three died and six dropped out for other reasons.

### Health states values

The observed number of participants, means and standard deviations of the SF-6D and NEI-VFQ are presented in Table 2. The SF-6D utility of the state was [BSE Mild-moderate; WSE Mild-moderate; 0.745] seems illogical ordered, and suggests an interaction between BSE and WSE. Patients with no visual loss in the BSE reported a lower mean (0.727) than patients with mild-moderate visual loss (0.745). The multilevel regression model revealed no such interaction effect (p = 0.953) between the BSE and WSE, so this effect was deleted from the models. The remaining parameters are presented in Table 3. The SF-6D utility of the health state with no visual loss in the BSE and mild-moderate visual loss in the WSE was estimated at 0.740, and the health state mild-moderate visual loss in the BSE and severe loss in the WSE was estimated at 0.684. The other health states had an intermediate position. Men had overall 0.04 point higher scores. The estimated values of the NEI VFQ-39 were 78.6 and 51.0 respectively. Older patients had overall 0.3 lower scores for each year they were older. Notable is the difference in parameter weight in the mild-moderate loss state between BSE and WSE. Where the NEI-VFQ-39 shows the BSE has a larger weight than the WSE (-16.2 vs. -4.4), the SF-6D shows the opposite direction with a smaller BSE weight than WSE weight (.008 vs. .033). As the parameters for the model are logical decrements, differences in modelled SF-6D utilities and scores of the NEI VFQ-39 are in the expected direction: better health states are associated with better scores ranging from 0.740 to 0.684 (Table 4).

**Table 3:** Multilevel regression parameters.

SF6D	Estimate [95% CI]	Significance
Intercept	.740 [.711, .769]	< .001
BSE–mild-moderate loss	-.008 [-.040, .025]	.645
WSE–mild moderate loss	-.033 [-.063, -.003]	.034
WSE–severe loss	-.049 [-.088, -.010]	.015
Male gender	.039 [.004, .075]	.031
Age	-.002 [-.005, .000]	.108
NEI-VFQ		
Intercept	78.618 [74.973, 82.263]	< .001
BSE–mild-moderate loss	-16.163 [-20.291, -12.035]	< .001
WSE–mild moderate loss	-4.433 [-8.559, -.306]	.035
WSE–severe loss	-11.474 [-16.540, -6.407]	< .001
Male gender	2.856 [-1.293, 7.004]	.176
Age	-.342 [-.654, -.029]	.032

**Table 4:** Quality of life utilities [95% CI] per health state.

		WSE			
	BSE	No visual loss	Mild-moderate	Severe	Mean
**SF-6D**	No visual loss	.740 [.711, .769]	.707 [.680, .734]	.691 [.658, .725]	.713 [.690, .735]
*n* = 309	Mild-moderate		.699 [.666, .732]	.684 [.649, .718]	.691 [.662, .721]
	Mean	.740 [.711, .769]	.703 [.678, .728]	.687 [.658, .717]	.704 [.685, .725]
**NEI-VFQ**	No visual loss	78.6 [75.0, 82.3]	74.2 [70.9, 77.5]	67.1 [63.0, 71.3]	73.3 [70.6, 76.0]
*n* = 341	Mild-moderate		58.0 [53.9, 62.2]	51.0 [46.8, 55.2]	54.5 [50.9, 58.1]
	Mean	78.6 [75.0, 82.3]	66.1 [63.0, 69.2]	59.1 [55.4, 62.7]	65.8 [63.4, 68.2]

Estimates at mean age and for women.

Abbreviations: BSE = better seeing eye; WSE = worse seeing eye.

no visual loss (≥20/40), mild-moderate visual loss (<20/40–>20/200) and severe visual loss (≤20/200).

## Discussion

### Principal findings

Societal utility values are provided for ARMD patients using the generic QoL instrument SF-6D for visual acuity health states based on both BSE and WSE.

### Imbedding in the literature

One of the implications of this research is that it can be confirmed that visual acuity changes in the WSE should be taken into account in health economic evaluations. Simply stating that changes in the WSE have no effect on QoL is not supported by the results of this study: difference in visual acuity in the WSE does have a relation with utility. This implies that setting utility changes for the WSE to zero in health economic models will result in an underestimation of the cost effectiveness of effective treatments. Additionally, assuming that effects in the WSE are as valuable as in the BSE is an unjust simplification as the results of the multilevel regression of the SF-6D shows an even higher value for the WSE. Our finding stresses the need for realistic utilities for both changes in the BSE as well as for changes in the WSE.

It has been suggested that, in order to be sensitive in ARMD, a utility measure should have a dimension which is directly linked to vision [18]. Our findings state that the SF-6D is sensitive in ARMD, although it has no such direct link with vision. On one hand, it can be assumed that the SF-6D underestimates the effects. On the other hand, the use of the generic SF-6D avoids the difficulty associated with disease-specific measures, like the focused effect, scale problems, the exclusion of side effects and comorbidities [13]. All those complications tend to overestimate the effects. Indeed, as theory predicts, our modelled range of the effects [0.740, 0.684] is smaller and at a lower place at the scale than Finger et al. measured with the disease-specific instrument VisQoL [0.95, 0.84] [8]. Moreover, the place on the scale of our SF-6D is in line with the EQ-5D range found by Finger et al. [0.70, 0.67]. The range of the EQ-5D values of Finger et al. is a smaller, which might reflect the better sensitivity of the SF-6D in the higher region of the utility scale, as compared to the EQ-5D [20]. Indeed Finger et al. did not find statistical significant results when modeling the EQ-5D utilities to visual acuity, while we did find a relation between visual acuity and the utilities of the SF-6D. Therefore, we argue that our results represent a conservative estimate of differences between health states of visual acuity, without the complication that utilities estimated from disease-specific instruments bring.

It is too early to conclude that the disease-specific utilities of the VisQoL provide more sensitive outcomes than the SF-6D utilities reported in the present paper, as the variation in the utilities of both instruments are in the extreme health states.

### Limitations

A limitation in our study is that the raw scores suggest that some health states have imprecise values, as the ordering is illogical. In the observed means (Table 2) we found an inconsistency within the group of patients with a mild-moderate visual loss in the WSE. Patients with no visual loss in the BSE reported a lower mean (0.727) than patients with mild-moderate visual loss (0.745). By using the multilevel model, these flaws are modelled out, but the model also reduces the range. This contributes to the idea that our results are an underestimation the real effects. More values per health states from further research could provide more precise values.

When the study was initiated, the main focus was on the study eye, measured with an ETDRS LogMar chart. As the non-treatment eye was measured according to daily clinical practice, the Snellen chart was used. This difference in approach might complicate comparability of the visual acuity between both eyes. We think we were able to compensate for this difference, as we accounted for the known measurement errors and sensitivity of the used visual acuity charts. When no distinction could be made between the BSE and WSE, eyes were framed as equal visual acuity.

A limitation in the generalizability of our results is that we did not include patients with worse than 20/200 visual acuity in their BSE. So values from this investigation need consideration if and when many patients belong to that population.

It might be suggested that the reduction of the number of health states to five health states may be too rigorous. However, a larger number of health states will make the estimation of stable transition- and cost-parameters more complex. In economic modeling, results of studies with more health states than the five states used here are likely to be rearranged to fewer health states anyway. Of course in larger patient cohorts it would relatively easy to increase the number of separate health states.

### Conclusion

Societal utility values are provided for ARMD patients using the generic QoL instrument SF-6D for visual acuity health states based on both BSE and WSE. The range of the values is smaller than that of the utilities elicited with the disease-specific instrument VisQoL, but the utilities are placed on a more realistic position on the utility scale. Furthermore, the utilities of the SF-6D avoid the problem associated with the interpretation of disease-specific utilities.
